# (*E*)-2-Meth­oxy-*N*′-(2,4,6-trihy­droxy­benzyl­idene)benzohydrazide

**DOI:** 10.1107/S1600536813001748

**Published:** 2013-01-23

**Authors:** Muhammad Taha, M. Syukri Baharudin, Nor Hadiani Ismail, Syed Adnan Ali Shah, Sammer Yousuf

**Affiliations:** a Atta-ur-Rahman Institute for Natural Product Discovery, Universiti Teknologi MARA (UiTM), Puncak Alam Campus, 42300 Bandar Puncak Alam, Selangor D. E. Malaysia; b Faculty of Applied Science, Universiti Teknologi MARA (UiTM), 40450 Shah Alam, Selangor D. E. Malaysia; c Department of Pharmacology and Chemistry, Faculty of Pharmacy, Universiti Teknologi MARA (UiTM), Puncak Alam Campus, 42300 Puncak Alam, Selangor D. E., Malaysia; d H.E.J. Research Institute of Chemistry, International Center for Chemical and Biological Sciences, University of Karachi, Karachi 75270, Pakistan

## Abstract

In the title hydrazone derivative, C_15_H_14_N_2_O_5_, the benzene rings are twisted by 7.55 (8)° with respect to each other. The azomethine double bond adopts an *E* conformation. The mol­ecular structure is stabilized by intra­molecular O—H⋯N and N—H⋯O hydrogen bonds, generating *S*6 ring motifs. In the crystal, mol­ecules are linked into a three-dimensional network by O—H⋯O hydrogen bonds.

## Related literature

For applications and biological activity of Schiff bases, see: Khan *et al.* (2011 ▶, 2012 ▶); Rada & Leto (2008 ▶); Almasirad *et al.* (2006 ▶). For related structures, see: Taha *et al.* (2012 ▶); Shen *et al.* (2012 ▶).
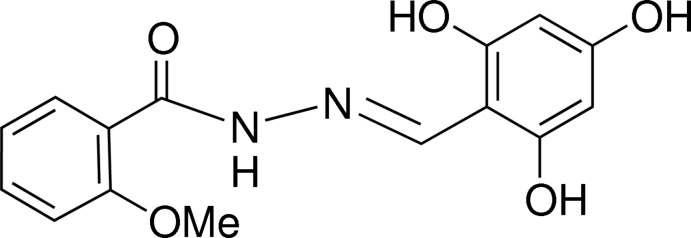


## Experimental

### 

#### Crystal data

C_15_H_14_N_2_O_5_
*M* *_r_* = 302.28
Orthorhombic, 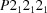

*a* = 6.4580 (4) Å
*b* = 13.4772 (8) Å
*c* = 16.3169 (9) Å
*V* = 1420.15 (14) Å^3^
*Z* = 4
Mo *K*α radiation
μ = 0.11 mm^−1^
*T* = 273 K
0.34 × 0.23 × 0.21 mm

#### Data collection

Bruker SMART APEX CCD area-detector diffractometer
Absorption correction: multi-scan (*SADABS*; Bruker, 2000 ▶) *T*_min_ = 0.964, *T*_max_ = 0.978
8414 measured reflections
2643 independent reflections
2481 reflections with *I* > 2σ(*I*)
*R*_int_ = 0.019

#### Refinement

*R*[*F* ^2^ > 2σ(*F* ^2^)] = 0.030
*wR*(*F* ^2^) = 0.080
*S* = 1.07
2643 reflections
216 parameters
H atoms treated by a mixture of independent and constrained refinement
Δρ_max_ = 0.13 e Å^−3^
Δρ_min_ = −0.13 e Å^−3^

### 

Data collection: *SMART* (Bruker, 2000 ▶); cell refinement: *SAINT* (Bruker, 2000 ▶); data reduction: *SAINT*; program(s) used to solve structure: *SHELXS97* (Sheldrick, 2008 ▶); program(s) used to refine structure: *SHELXL97* (Sheldrick, 2008 ▶); molecular graphics: *SHELXTL* (Sheldrick, 2008 ▶); software used to prepare material for publication: *SHELXTL*, *PARST* (Nardelli, 1995 ▶) and *PLATON* (Spek, 2009 ▶).

## Supplementary Material

Crystal structure: contains datablock(s) global, I. DOI: 10.1107/S1600536813001748/rz5037sup1.cif

Structure factors: contains datablock(s) I. DOI: 10.1107/S1600536813001748/rz5037Isup2.hkl

Supplementary material file. DOI: 10.1107/S1600536813001748/rz5037Isup3.cml

Additional supplementary materials:  crystallographic information; 3D view; checkCIF report

## Figures and Tables

**Table 1:** Hydrogen-bond geometry (Å, °)

*D*—H⋯*A*	*D*—H	H⋯*A*	*D*⋯*A*	*D*—H⋯*A*
O1—H1*A*⋯N1	0.84 (3)	1.88 (2)	2.6243 (18)	147 (2)
N2—H2*A*⋯O5	0.851 (19)	1.923 (19)	2.5981 (18)	135.4 (17)
O3—H2*B*⋯O4^i^	0.86 (2)	1.79 (2)	2.6452 (17)	175 (2)
O2—H3*A*⋯O1^ii^	0.93 (3)	1.92 (3)	2.8513 (18)	172 (3)

Symmetry codes: (i) 
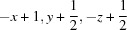
; (ii) 
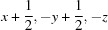
.

**Fig. 1.**
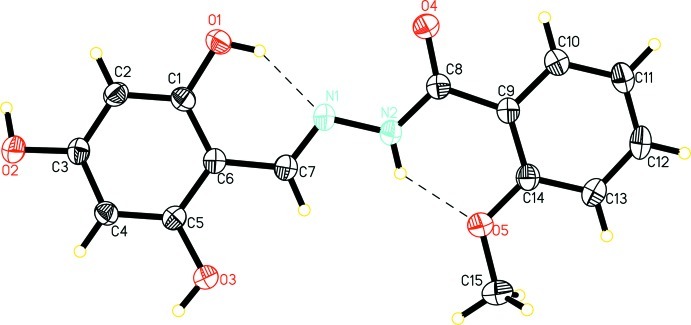
The molecular structure of the title compound with displacement ellipsoids drawn at 30% probability level. Intramolecular hydrogen bonds are shown as dashed lines.

**Fig. 2.**
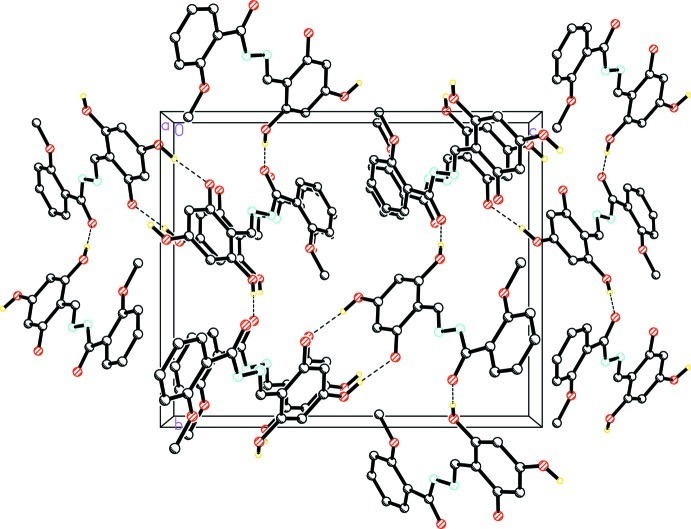
Crystal packing of the title compound viewed down the a axis. Only hydrogen atoms involved in hydrogen bonding are shown.

## supplementary crystallographic information

### Comment

Hydrazone derivatives represent an important class of organic compounds. Due to their biological activities (Khan *et al.*, 2011, 2012; Rada & Leto, 2008; Almasirad *et al.*, 2006) the research for this class of compounds is an area of great interest. The title compound is a hydrazone derivatives synthesized as a part of our ongoing research to establish a library of bioactive hydrazone derivatives.

The structure of the title compound (Fig. 1) is similar to that of the previously published compound *N*'-(3,4-dihydroxybenzylidene)-2- methoxybenzohydrazide (Shen *et al.*, 2012) with the difference that the 3,4-dihydroxy benzene ring is replaced by a 2,4,6-trihydroxy benzene ring (C1–C6). The dihedral angle between the two benzene rings is 7.55 (8)°. The bond lengths and angles were found to be similar to those observed in structurally related benzohydrazide derivatives (Taha *et al.*, 2012; Shen *et al.*, 2012). Intramolecular O1—H1A···N1 and N2—H2A···O5 hydrogen bonds play an important role to stabilize the *E* configuration of the azomethine olefinic bond (Table 1). The crystal structure (Fig. 2) is stabilized by intermolecular O3—H2B···O4 and O2—H3A···O1 interactions to form a three-dimensional network.

### Experimental

The title compound was synthesized by refluxing a mixture of 2-methoxybenzohydrazide (0.332 g, 2 mmol) and 2,4,6-trihydroxy-5-methoxybenzaldehyde (0.304 g, 2 mmol) in methanol along with a catalytical amount of acetic acid for 3 h. The progress of reaction was monitored by TLC. After completion of the reaction, the solvent was evaporated by vacuum to afford the crude product which was recrystallized by dissolving in methanol at room temperature. Needle-shaped crystals were obtained on slow evaporation of the solvent (0.496 g, 82% yield). All chemicals were purchased by Sigma Aldrich, Germany.

### Refinement

H atoms on methyl, phenyl and methine carbon atoms were positioned geometrically with C—H = 0.96 Å (CH_3_) and 0.93 Å (CH) and constrained to ride on their parent atoms with *U*_iso_(H)= 1.5*U*_eq_(CH_3_) or 1.2*U*_eq_(CH). The H atoms on the nitrogen (N–H= 0.835 (17) Å) and oxygen (O–H= 0.84 (2)–0.93(2 Å) atoms were located in a difference Fourier map and refined isotropically. A rotating group model was applied to the methyl group.

### Crystal data

**Table:**

C_15_H_14_N_2_O_5_	*F*(000) = 632
*M**_r_* = 302.28	*D*_x_ = 1.414 Mg m^−^^3^
Orthorhombic, *P*2_1_2_1_2_1_	Mo *K*α radiation, λ = 0.71073 Å
Hall symbol: P 2ac 2ab	Cell parameters from 3988 reflections
*a* = 6.4580 (4) Å	θ = 2.5–27.8°
*b* = 13.4772 (8) Å	µ = 0.11 mm^−^^1^
*c* = 16.3169 (9) Å	*T* = 273 K
*V* = 1420.15 (14) Å^3^	Block, colourless
*Z* = 4	0.34 × 0.23 × 0.21 mm

### Data collection

**Table:**

Bruker SMART APEX CCD area-detector diffractometer	2643 independent reflections
Radiation source: fine-focus sealed tube	2481 reflections with *I* > 2σ(*I*)
Graphite monochromator	*R*_int_ = 0.019
ω scan	θ_max_ = 25.5°, θ_min_ = 2.0°
Absorption correction: multi-scan (*SADABS*; Bruker, 2000)	*h* = −6→7
*T*_min_ = 0.964, *T*_max_ = 0.978	*k* = −16→16
8414 measured reflections	*l* = −19→19

### Refinement

**Table:**

Refinement on *F*^2^	Primary atom site location: structure-invariant direct methods
Least-squares matrix: full	Secondary atom site location: difference Fourier map
*R*[*F*^2^ > 2σ(*F*^2^)] = 0.030	Hydrogen site location: inferred from neighbouring sites
*wR*(*F*^2^) = 0.080	H atoms treated by a mixture of independent and constrained refinement
*S* = 1.07	*w* = 1/[σ^2^(*F*_o_^2^) + (0.0444*P*)^2^ + 0.1141*P*] where *P* = (*F*_o_^2^ + 2*F*_c_^2^)/3
2643 reflections	(Δ/σ)_max_ < 0.001
216 parameters	Δρ_max_ = 0.13 e Å^−^^3^
0 restraints	Δρ_min_ = −0.13 e Å^−^^3^

### Special details

**Table:**

Geometry. All e.s.d.'s (except the e.s.d. in the dihedral angle between two l.s. planes) are estimated using the full covariance matrix. The cell e.s.d.'s are taken into account individually in the estimation of e.s.d.'s in distances, angles and torsion angles; correlations between e.s.d.'s in cell parameters are only used when they are defined by crystal symmetry. An approximate (isotropic) treatment of cell e.s.d.'s is used for estimating e.s.d.'s involving l.s. planes.
Refinement. Refinement of *F*^2^ against ALL reflections. The weighted *R*-factor *wR* and goodness of fit *S* are based on *F*^2^, conventional *R*-factors *R* are based on *F*, with *F* set to zero for negative *F*^2^. The threshold expression of *F*^2^ > σ(*F*^2^) is used only for calculating *R*-factors(gt) *etc*. and is not relevant to the choice of reflections for refinement. *R*-factors based on *F*^2^ are statistically about twice as large as those based on *F*, and *R*- factors based on ALL data will be even larger.

### Fractional atomic coordinates and isotropic or equivalent isotropic displacement parameters (Å^2^)

**Table:**

	*x*	*y*	*z*	*U*_iso_*/*U*_eq_	
O1	0.5330 (2)	0.22125 (9)	0.11606 (8)	0.0543 (3)	
O2	1.1070 (2)	0.40655 (9)	0.02009 (8)	0.0578 (3)	
H3A	1.091 (5)	0.3604 (19)	−0.0222 (16)	0.100 (8)*	
O3	0.6905 (2)	0.53380 (9)	0.23545 (7)	0.0511 (3)	
H2B	0.785 (3)	0.5781 (16)	0.2396 (12)	0.065 (7)*	
O4	0.0021 (2)	0.16307 (8)	0.25569 (7)	0.0518 (3)	
O5	−0.0653 (2)	0.42748 (8)	0.38286 (8)	0.0578 (3)	
N1	0.2953 (2)	0.30535 (10)	0.22647 (8)	0.0421 (3)	
N2	0.1262 (2)	0.31688 (11)	0.27673 (8)	0.0436 (3)	
H2A	0.111 (3)	0.3735 (14)	0.2990 (11)	0.054 (5)*	
C1	0.6522 (2)	0.30443 (11)	0.12072 (9)	0.0403 (3)	
C2	0.8209 (3)	0.31059 (12)	0.06945 (10)	0.0447 (4)	
H2C	0.8508	0.2594	0.0331	0.054*	
C3	0.9454 (3)	0.39399 (11)	0.07272 (9)	0.0419 (4)	
C4	0.9087 (2)	0.46946 (11)	0.12866 (9)	0.0410 (3)	
H4A	0.9973	0.5238	0.1318	0.049*	
C5	0.7383 (2)	0.46267 (10)	0.17972 (9)	0.0382 (3)	
C6	0.6031 (2)	0.38110 (11)	0.17590 (9)	0.0377 (3)	
C7	0.4203 (3)	0.37914 (12)	0.22646 (9)	0.0411 (3)	
H7A	0.3922	0.4331	0.2602	0.049*	
C8	−0.0127 (2)	0.24489 (11)	0.28886 (9)	0.0385 (3)	
C9	−0.1900 (2)	0.26844 (11)	0.34441 (9)	0.0376 (3)	
C10	−0.3409 (3)	0.19578 (12)	0.35197 (10)	0.0448 (4)	
H10A	−0.3256	0.1368	0.3231	0.054*	
C11	−0.5130 (3)	0.20830 (14)	0.40098 (10)	0.0521 (4)	
H11A	−0.6111	0.1581	0.4055	0.063*	
C12	−0.5376 (3)	0.29535 (14)	0.44283 (10)	0.0550 (5)	
H12A	−0.6537	0.3043	0.4758	0.066*	
C13	−0.3922 (3)	0.37023 (14)	0.43680 (10)	0.0512 (4)	
H13A	−0.4119	0.4295	0.4650	0.061*	
C14	−0.2165 (3)	0.35718 (12)	0.38868 (9)	0.0431 (4)	
C15	−0.0887 (4)	0.52036 (14)	0.42409 (13)	0.0725 (6)	
H15A	0.0214	0.5642	0.4082	0.109*	
H15B	−0.0837	0.5099	0.4823	0.109*	
H15C	−0.2194	0.5494	0.4096	0.109*	
H1A	0.432 (4)	0.2267 (18)	0.1484 (15)	0.094 (9)*	

### Atomic displacement parameters (Å^2^)

**Table:**

	*U*^11^	*U*^22^	*U*^33^	*U*^12^	*U*^13^	*U*^23^
O1	0.0576 (8)	0.0517 (7)	0.0535 (7)	−0.0166 (6)	0.0105 (6)	−0.0109 (5)
O2	0.0546 (8)	0.0572 (7)	0.0615 (7)	−0.0047 (6)	0.0220 (7)	−0.0072 (6)
O3	0.0522 (8)	0.0449 (7)	0.0562 (7)	−0.0059 (6)	0.0124 (6)	−0.0104 (5)
O4	0.0498 (7)	0.0414 (6)	0.0640 (7)	0.0035 (5)	0.0084 (6)	−0.0035 (5)
O5	0.0560 (8)	0.0481 (6)	0.0692 (8)	−0.0065 (6)	0.0153 (7)	−0.0140 (6)
N1	0.0348 (7)	0.0485 (7)	0.0429 (7)	0.0014 (6)	0.0043 (6)	0.0031 (6)
N2	0.0360 (7)	0.0438 (7)	0.0509 (8)	0.0004 (6)	0.0083 (6)	−0.0015 (6)
C1	0.0416 (9)	0.0404 (7)	0.0388 (7)	−0.0034 (7)	−0.0035 (7)	0.0019 (6)
C2	0.0500 (10)	0.0429 (8)	0.0411 (8)	0.0020 (8)	0.0065 (7)	−0.0041 (6)
C3	0.0387 (9)	0.0456 (8)	0.0416 (8)	0.0031 (7)	0.0052 (7)	0.0058 (6)
C4	0.0411 (9)	0.0375 (7)	0.0444 (8)	−0.0031 (7)	0.0017 (7)	0.0022 (6)
C5	0.0405 (8)	0.0375 (7)	0.0365 (7)	0.0049 (7)	−0.0005 (7)	0.0021 (6)
C6	0.0362 (8)	0.0416 (8)	0.0354 (7)	0.0020 (7)	−0.0004 (7)	0.0043 (6)
C7	0.0388 (9)	0.0448 (8)	0.0396 (8)	0.0021 (7)	0.0009 (7)	0.0021 (6)
C8	0.0355 (8)	0.0398 (8)	0.0401 (7)	0.0060 (7)	−0.0030 (7)	0.0061 (6)
C9	0.0341 (8)	0.0421 (8)	0.0366 (7)	0.0034 (6)	−0.0015 (6)	0.0057 (6)
C10	0.0418 (9)	0.0471 (8)	0.0453 (8)	−0.0014 (7)	−0.0017 (7)	0.0050 (7)
C11	0.0427 (10)	0.0657 (11)	0.0480 (9)	−0.0100 (9)	0.0030 (8)	0.0080 (8)
C12	0.0410 (10)	0.0777 (12)	0.0462 (9)	0.0026 (9)	0.0109 (8)	0.0080 (8)
C13	0.0520 (11)	0.0583 (10)	0.0433 (8)	0.0049 (9)	0.0070 (8)	−0.0027 (7)
C14	0.0408 (9)	0.0484 (8)	0.0403 (8)	0.0006 (7)	0.0002 (7)	0.0037 (7)
C15	0.0877 (16)	0.0541 (11)	0.0757 (12)	−0.0109 (11)	0.0154 (12)	−0.0200 (9)

### Geometric parameters (Å, º)

**Table:**

O1—C1	1.3622 (18)	C4—H4A	0.9300
O1—H1A	0.84 (3)	C5—C6	1.405 (2)
O2—C3	1.362 (2)	C6—C7	1.440 (2)
O2—H3A	0.93 (3)	C7—H7A	0.9300
O3—C5	1.3569 (18)	C8—C9	1.494 (2)
O3—H2B	0.86 (2)	C9—C10	1.387 (2)
O4—C8	1.2321 (19)	C9—C14	1.408 (2)
O5—C14	1.364 (2)	C10—C11	1.379 (2)
O5—C15	1.429 (2)	C10—H10A	0.9300
N1—C7	1.281 (2)	C11—C12	1.367 (3)
N1—N2	1.3742 (18)	C11—H11A	0.9300
N2—C8	1.336 (2)	C12—C13	1.382 (3)
N2—H2A	0.851 (19)	C12—H12A	0.9300
C1—C2	1.376 (2)	C13—C14	1.391 (2)
C1—C6	1.407 (2)	C13—H13A	0.9300
C2—C3	1.383 (2)	C15—H15A	0.9600
C2—H2C	0.9300	C15—H15B	0.9600
C3—C4	1.387 (2)	C15—H15C	0.9600
C4—C5	1.384 (2)		
			
C1—O1—H1A	109.3 (17)	C6—C7—H7A	119.0
C3—O2—H3A	107.3 (17)	O4—C8—N2	122.19 (14)
C5—O3—H2B	112.6 (14)	O4—C8—C9	121.05 (14)
C14—O5—C15	120.00 (14)	N2—C8—C9	116.75 (13)
C7—N1—N2	114.39 (13)	C10—C9—C14	117.96 (14)
C8—N2—N1	122.66 (13)	C10—C9—C8	116.26 (13)
C8—N2—H2A	120.7 (14)	C14—C9—C8	125.78 (14)
N1—N2—H2A	116.6 (14)	C11—C10—C9	122.09 (16)
O1—C1—C2	117.59 (14)	C11—C10—H10A	119.0
O1—C1—C6	120.85 (14)	C9—C10—H10A	119.0
C2—C1—C6	121.56 (14)	C12—C11—C10	119.24 (16)
C1—C2—C3	119.07 (14)	C12—C11—H11A	120.4
C1—C2—H2C	120.5	C10—C11—H11A	120.4
C3—C2—H2C	120.5	C11—C12—C13	120.80 (16)
O2—C3—C2	121.47 (14)	C11—C12—H12A	119.6
O2—C3—C4	117.05 (14)	C13—C12—H12A	119.6
C2—C3—C4	121.47 (14)	C12—C13—C14	120.14 (16)
C5—C4—C3	118.93 (14)	C12—C13—H13A	119.9
C5—C4—H4A	120.5	C14—C13—H13A	119.9
C3—C4—H4A	120.5	O5—C14—C13	122.37 (15)
O3—C5—C4	122.54 (14)	O5—C14—C9	117.88 (14)
O3—C5—C6	116.16 (13)	C13—C14—C9	119.75 (15)
C4—C5—C6	121.29 (13)	O5—C15—H15A	109.5
C5—C6—C1	117.57 (13)	O5—C15—H15B	109.5
C5—C6—C7	119.87 (13)	H15A—C15—H15B	109.5
C1—C6—C7	122.55 (14)	O5—C15—H15C	109.5
N1—C7—C6	122.06 (14)	H15A—C15—H15C	109.5
N1—C7—H7A	119.0	H15B—C15—H15C	109.5
			
C7—N1—N2—C8	−175.22 (14)	N1—N2—C8—O4	0.1 (2)
O1—C1—C2—C3	−179.90 (15)	N1—N2—C8—C9	−179.32 (13)
C6—C1—C2—C3	0.4 (2)	O4—C8—C9—C10	−3.9 (2)
C1—C2—C3—O2	−176.07 (15)	N2—C8—C9—C10	175.56 (14)
C1—C2—C3—C4	2.6 (2)	O4—C8—C9—C14	176.47 (15)
O2—C3—C4—C5	175.90 (14)	N2—C8—C9—C14	−4.1 (2)
C2—C3—C4—C5	−2.8 (2)	C14—C9—C10—C11	0.0 (2)
C3—C4—C5—O3	−179.30 (14)	C8—C9—C10—C11	−179.71 (14)
C3—C4—C5—C6	0.1 (2)	C9—C10—C11—C12	0.8 (2)
O3—C5—C6—C1	−177.87 (13)	C10—C11—C12—C13	−0.3 (3)
C4—C5—C6—C1	2.7 (2)	C11—C12—C13—C14	−0.9 (3)
O3—C5—C6—C7	3.1 (2)	C15—O5—C14—C13	−2.9 (2)
C4—C5—C6—C7	−176.29 (13)	C15—O5—C14—C9	177.58 (16)
O1—C1—C6—C5	177.32 (14)	C12—C13—C14—O5	−177.81 (16)
C2—C1—C6—C5	−2.9 (2)	C12—C13—C14—C9	1.7 (2)
O1—C1—C6—C7	−3.7 (2)	C10—C9—C14—O5	178.31 (14)
C2—C1—C6—C7	176.02 (15)	C8—C9—C14—O5	−2.0 (2)
N2—N1—C7—C6	−178.41 (13)	C10—C9—C14—C13	−1.2 (2)
C5—C6—C7—N1	−178.30 (14)	C8—C9—C14—C13	178.45 (14)
C1—C6—C7—N1	2.8 (2)		

### Hydrogen-bond geometry (Å, º)

**Table:**

*D*—H···*A*	*D*—H	H···*A*	*D*···*A*	*D*—H···*A*
O1—H1*A*···N1	0.84 (3)	1.88 (2)	2.6243 (18)	147 (2)
N2—H2*A*···O5	0.851 (19)	1.923 (19)	2.5981 (18)	135.4 (17)
O3—H2*B*···O4^i^	0.86 (2)	1.79 (2)	2.6452 (17)	175 (2)
O2—H3*A*···O1^ii^	0.93 (3)	1.92 (3)	2.8513 (18)	172 (3)

Symmetry codes: (i) −*x*+1, *y*+1/2, −*z*+1/2; (ii) *x*+1/2, −*y*+1/2, −*z*.
